# Effective Network Size Predicted From Simulations of Pathogen Outbreaks Through Social Networks Provides a Novel Measure of Structure-Standardized Group Size

**DOI:** 10.3389/fvets.2018.00071

**Published:** 2018-05-03

**Authors:** Collin M. McCabe, Charles L. Nunn

**Affiliations:** ^1^Department of Human Evolutionary Biology, Harvard University, Cambridge, MA, United States; ^2^Division of Infectious Diseases and Global Health, Department of Medicine, Duke University, Durham, NC, United States; ^3^Department of Evolutionary Anthropology, Duke University, Durham, NC, United States; ^4^Triangle Center for Evolutionary Medicine (TriCEM), Durham, NC, United States

**Keywords:** social network analysis, compartmental modeling, simulation modeling, group size, parasites, disease ecology, disease outbreaks

## Abstract

The transmission of infectious disease through a population is often modeled assuming that interactions occur randomly in groups, with all individuals potentially interacting with all other individuals at an equal rate. However, it is well known that pairs of individuals vary in their degree of contact. Here, we propose a measure to account for such heterogeneity: effective network size (ENS), which refers to the size of a maximally complete network (i.e., unstructured, where all individuals interact with all others equally) that corresponds to the outbreak characteristics of a given heterogeneous, structured network. We simulated susceptible-infected (SI) and susceptible-infected-recovered (SIR) models on maximally complete networks to produce idealized outbreak duration distributions for a disease on a network of a given size. We also simulated the transmission of these same diseases on random structured networks and then used the resulting outbreak duration distributions to predict the ENS for the group or population. We provide the methods to reproduce these analyses in a public R package, “enss.” Outbreak durations of simulations on randomly structured networks were more variable than those on complete networks, but tended to have similar mean durations of disease spread. We then applied our novel metric to empirical primate networks taken from the literature and compared the information represented by our ENSs to that by other established social network metrics. In AICc model comparison frameworks, group size and mean distance proved to be the metrics most consistently associated with ENS for SI simulations, while group size, centralization, and modularity were most consistently associated with ENS for SIR simulations. In all cases, ENS was shown to be associated with at least two other independent metrics, supporting its use as a novel metric. Overall, our study provides a proof of concept for simulation-based approaches toward constructing metrics of ENS, while also revealing the conditions under which this approach is most promising.

## Introduction

Theoretical models allow us to make sense of complex phenomena by applying a set of simplifying assumptions. In many cases, however, empirical observations of the phenomena do not conform to these assumptions. Understanding how observations compare to their theoretical ideals is thus critical to the interpretation of any such model. Within biology, one of the earliest attempts to compare observations to their theoretical ideals was the work of Wright on effective population size (1). Effective population size models take an observed population with a certain amount of genetic diversity and predict the size of an idealized population under the assumptions of Fisher–Wright populations that groups are of finite and fixed sizes, individuals mate randomly, and generations do not overlap (2–4). The generalizability of effective population size allows biologists to compare populations, which is useful in many contexts, including wildlife management and conservation policies (5).

Infectious disease represents another phenomenon in which the concept of an idealized population is useful. As with effective population size, a set of simplifying assumptions exist that can be repurposed to formulate theoretically idealized populations, given an observed population. Compartmental disease models aim to predict disease transmission by using assumptions similar to those in Fisher–Wright populations. For example, they assume that individuals transmit pathogens freely throughout the population, similar to the Fisher–Wright assumption of random mating (the free association assumption); individuals do not immigrate or emigrate, maintaining a Fisher–Wright constant population size; and there is no age structure within the population, with non-overlapping generations (6). However, these assumptions are rarely met in natural populations. As shown through early critiques of compartmental disease models (6) and more recently through the resurgence of social network studies, interactions are not random, but instead structured along social ties between specific individuals based on affiliative interactions, mating, and other social behaviors (7).

Here, we investigated how changes specifically to the free association assumption, through structuring in social networks, affect the time it takes for a disease to transmit through a population. To assess the deviation of an observed population from a theoretical ideal in disease transmission through structured groups, we must define what represents an idealized population and disease outbreak. Many ecological and environmental factors can affect group size and structure, including food distribution and predation. By “ideal,” we are referring to a perfect adherence to the assumption of free association. By “free association,” we are referring to the fact that all individuals have equal probabilities to interact with every other individual in the population, perfectly mirroring the mass action properties of traditional compartmental disease models at infinite population sizes.

**Figure 1:**
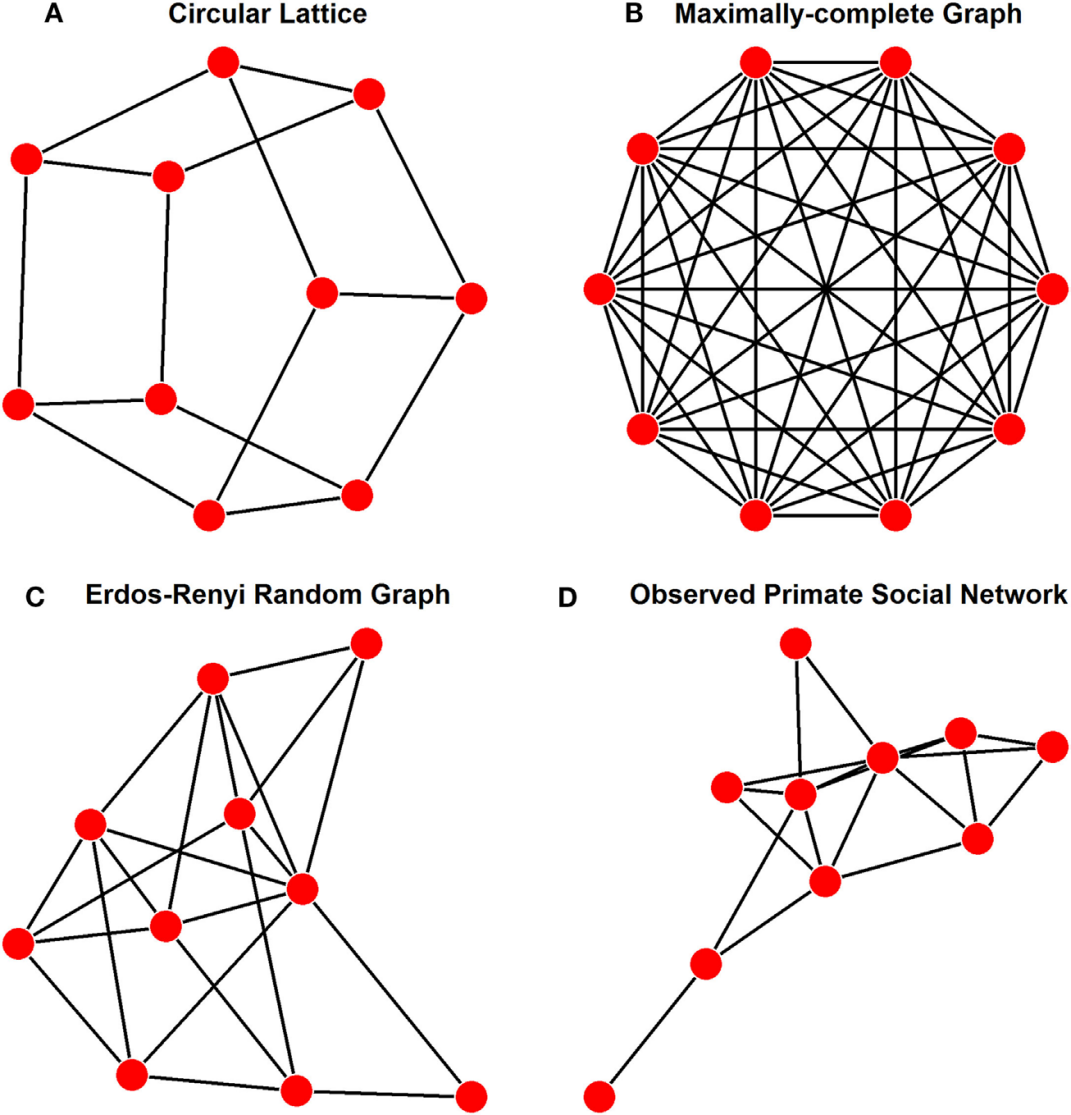
Examples of a population of 10 individuals showing various representative network structures, as discussed in the text. These different structures and their applications are **(A)** lattice structure as has been used in other network models for disease transmission, where ties are regular, but not exhaustively complete; **(B)** maximally complete structure as was used for our idealized networks with free association, each individual is connect to all other individuals in the population; **(C)** Erdõs-Rényi generation structure with every possible tie existing with a probability of 0.25, thus the number of ties in this graph are a quarter of those present in panel **(B)**; and **(D)** an example of an empirically observed network of social interactions among primates [*Pan troglodytes* (10)].

In a review of network modeling of epidemics, Keeling and Eames (8) suggest that a variety of idealized networks exist, depending on the end goal of the model. The purpose of our model is to allow free association between individuals in a social network. The earliest modeling of disease transmission through networks was conducted on lattices (9), with regularly structured connections between individuals (Figure 1A). However, lattices show too much deviation from the Fisher–Wright assumption of completely free and random association to be used as an idealized population. Instead, given the assumptions of basic compartmental models, the most fitting network arrangement to be used as an ideal is a maximally complete network, in which each individual has uniform ties to each other individual in the network, allowing for effectively free association among all nodes (Figure 1B).

As for the epidemiological model, either deterministic or stochastic models are used to model the transmission of disease. As we are aiming to simplify assumptions about the transmission of disease, deterministic models would provide more straightforward, less complicated views of disease transmission. However, deterministic models require an intimate knowledge of the dynamics of disease transmission within a population; unknown variables, such as the effect of social structure on outbreaks, make this sort of modeling impossible. Stochastic models, which are often more representative of real-world heterogeneity in disease transmission, allow for uncertainty in variables or dynamics by simulating many different, randomly selected values for important variables (11). For this reason, we employed stochastic models for our study.

Infectious diseases that are transmitted and maintained in populations can be modeled using a variety of epidemiological models. For instance, susceptible-infected (SI) models are useful for investigating the transmission of diseases caused by lifelong infections, where no recovery is possible; these models include specialized types of SI diseases, like sexually transmitted diseases, where transmission rates vary depending on which sex of individual is interacting. For following disease outbreaks through a population where recovery and resistance is possible, the simplest sufficient compartmental model would be a susceptible-infected-recovered (SIR) model, where susceptible individuals become infected from other infected individuals, but they will eventually be removed from the population of susceptible and infectious individuals, either recovering with full immunity to further infection or dying from the disease (which, for the purposes of our research, are functionally equivalent). To capture the large amount of variation among diverse types of diseases, and to be as relevant as possible to researchers studying a potentially wide variety of pathogens, we investigated SI and SIR models in this study using per contact transmission and recovery rates that were realistic but would still allow time for recovery or extinction in SIR models.

Previous work on determining the effective size of a network has focused on very specific aspects of network structure and has thus maintained a restricted conception of what constitutes an idealized network. In the only comparable epidemiological research on this topic, Caillaud et al. (12) proposed a measure of “epidemiological effective group size.” This metric considered the variation in sub-group size within a meta-population and the impact of this variation on the outbreak of a disease within the meta-population. By using maximally complete networks of sub-groups connected to other maximally complete sub-groups, the researchers calculated the likelihood of an epidemic outbreak throughout the meta-population based on the size of the index sub-group. Thus, Caillaud et al.’s (12) metric is essentially a novel measure of an invasion-specific critical community size needed to maintain an outbreak (13), which in addition to the previous measure, also takes variation in meta-population structure and sub-group size into account.

In addition, another notion of effective group size has been utilized in estimating the number of distinct cultural behaviors, or cultural richness, that is maintained within a human population. This approach was first theoretically developed by Henrich (14) using assumptions of even mixing for cultural transmission of multiple behaviors through a population; results of this analysis demonstrated that a decrease in the size of a population through geographic isolation could explain the loss of complex cultural behaviors among Tasmanian islanders. This method was further developed by Powell et al. (15) to incorporate spatial and temporal variability through estimates of population density and migration rates, respectively. Using this method, the researchers showed that the variability in human population density and migratory activity, resulting in “effective population sizes” for human groups, explained much of the geospatial distribution in cultural behaviors during the Late Pleistocene Epoch. These methods are closely related to those described in our study, in that each is using population structure to explain observed richness, either cultural or parasitic. However, the models for explaining observed richness of human behavior did not explicitly incorporate social network structure; this is the main contribution of our own method.

While our methods do incorporate the complexities of network structure in diseases transmission, we are omitting many other important factors of social structure and disease ecology. As just noted, social structure can have important impacts on the maintenance of cultural behaviors (14, 15), and cultural behaviors themselves have been shown to have significant impacts on disease transmission (16). In addition, several other factors can influence the structure and size of a group, including the relative despotism or tolerance of a group (17, 18), ecology (19), or resource availability (20). Our goal in omitting these factors from the following analyses is not to downplay any of their impacts on social network structure or disease transmission but rather to isolate the effect of social network structure on disease transmission using a simplified model.

The first specific aim of our study is to quantify the relationship between networks of various sizes and outbreak durations for diseases with and without immunity, and with variation in epidemiological parameters (focusing on variation in per contact probability of transmission). Here, we expect that infectious diseases transmitted through larger networks will show longer outbreak durations than disease transmitted through smaller networks (12). We investigate the relationship between group size and outbreak duration to provide a basis for calculating effective group size. The second specific aim is to generate randomly structured networks and to simulate disease transmission through those randomly structured networks to predict what sized maximally complete network would have the same outbreak duration; we call these the effective network size (ENS) of the social group. Just as we establish a relationship between outbreak duration and maximally complete network size to provide a baseline relationship between them, we use this same relationship between network size and outbreak duration to predict the ENS of randomly structured groups from the outbreak durations of their SI and SIR simulations. It is important to note that our measure of ENS will always be equal to or larger than the original group size, which differs significantly from effective population size, which is always equal to or smaller than the original group size. Among these simulations, we compare the accuracy and precision of using regression models to predict ENS from distributions of outbreak durations on the randomly structured networks. All of the methods described in this study can be easily replicated with a publicly accessible R package, enss, developed specifically for this study (https://www.github.com/collinmmccabe/enss), and the relevant functions for each step of the analysis are noted throughout the Section “Methods.”

Finally, as a proof of concept, we apply our new metric for representing disease transmission to a collection of primate networks (21). We then compare the information represented by ENS to other, more established network metrics to determine the novelty of our metric, as well as its associations with other metrics. The specific metrics that we investigate here are leading eigenvector modularity, mean distance, diameter, clustering coefficient, and eigenvector centralization, as were also investigated by Nunn and colleagues (22). In Data Sheet S1 in Supplementary Material (Supplementary Analysis), we also provide an example use case of ENS from these same primate species, comparing it to raw group size as a predictor of parasite richness.

## Methods

### Simulation and Regression of Disease Transmission on Maximally Complete Networks

To address the first aim of correlating idealized networks with disease transmission times, we generated maximally complete, unweighted, undirected networks for groups of size 3–200 in R, version 3.3.2 (23) with packages igraph (24), statnet (25), and functions that we developed and distribute in enss. We then simulated SI models (with a per contact transmission rate, β, of 0.10, and per capita interactions per day set at three times the group size) and SIR models (with an additional parameter, γ, or the daily recovery rate set at 0.10) to saturation or extinction (the points at which pathogens could not be transmitted further) on each of these networks 1,000 times. β and γ were both parameterized at 0.10, following previous disease simulations as described in Griffin and Nunn (26). For β, this value of 0.10 indicated that for every interaction between a susceptible and an infected individual, there was a 10% probability that the susceptible would become infected; for γ, the 0.10 indicated that per daily timestep, each infected individual had a 10% probability of recovering. These methods are available through R package enss as functions “clust_sim_SI” and “clust_sim_SIR,” respectively. Although we chose to focus our efforts by using unweighted networks and testing only one value for β, we also present analyses that investigate the effects of incorporating weighted ties and varying values of β.

Per capita social interaction rates per day were chosen arbitrarily to be at a rate of three interactions per individual per day in the analyses presented here. This means that for a group of size 10, 30 random interactions were independently chosen from the set of all available interactions between individuals in the group, which was then repeated for each daily timestep of the model’s simulation. Days and per capita interaction rates per day were used as familiar, but ultimately arbitrary demarcations of time in our models so that “outbreak duration” could be measured in a uniform manner.

Our algorithms for disease transmission on networks took place in multiple stages. The first stage involved generating and recording social networks as edgelists, where each social tie between two individuals is recoded as its own row of data. This method can be replicated using enss function “gen_max.” We also tracked the infection status of each node, or individual in the network, as susceptible, infected, or recovered. From among these nodes, one was selected as an index case and was infected at the outset of the simulation.

Following previous disease simulations from Griffin and Nunn (26), we then selected consecutive random edges, or social ties between individuals, to determine whether the disease could be transmitted from one node to another (with a probability β of transmission for each interaction); the number of edges that were selected depended on the per capita interactions per day, or 3N, and the number of individuals in the network (ranging from 3 to 200). So, for a network of 10 individuals, we chose 30 random edges each day, allowing for the possibility of repeated sampling of social ties. For each of these edges, we checked whether transmission was possible; in our models, the only opportunity for disease transmission was the case where an edge connected an infected individual with a susceptible one, ignoring any directionality in the interaction. Each edge over which transmission was possible resulted in an actual transmission event (where the susceptible individual becomes infected) with probability β = 0.10, as described above; this would result in 10% of interactions between susceptibles and infecteds resulting in transmission. After all random edges had been considered for a day, each infected individual in SIR models randomly recovered with a probability γ. The simulation then moved to the next day, and only stopped when the criteria for simulation completion were met. No maximum duration was set for either SI or SIR models (because these models would eventually reach either saturation or extinction).

We also considered transmission models where each tie in a graph was sampled once per day, rather than randomly in proportion to the number of nodes, because the number of edges in networks grows exponentially with the number of nodes (Equation S1 in Supplementary Material), and per capita interaction rates in large networks would be less likely to represent a given tie than in a smaller network. We call this the “alternative model,” and give results in Figure S1 in Supplementary Material. These methods are available in enss as functions “clust_sim_SI_unif” and “clust_sim_SIR_unif.” In addition, we also considered transmission models where ties were weighted. In such models, ties with greater weights, or intensity of interaction between two individuals, were sampled more often than lesser weighted ties. In these models, ties were still sampled randomly at the per capita interaction rate per day, but the likelihood of sampling a given tie was proportional to its weight. This model is called the “weighted model” in analyses that follow. These methods are also available in enss as functions “clust_sim_SI_w” and “clust_sim_SIR_w.”

We recorded the number of days until the simulation ended as “outbreak duration.” For SI models, simulations ended at saturation, defined as the point at which all individuals had transitioned from susceptible to infected. For SIR models, simulations ended at extinction, defined as the point at which no infected individuals were present in the population, either because all susceptible individuals had been infected and subsequently recovered, or because all infected individuals recovered without being able to sustain further transmission to remaining susceptible nodes. We then found a line of best fit through the results for each epidemiological model, using regression models to predict network sizes from outbreak durations. The output for these linear models can be generated in enss with functions “predict_SI_max” and “predict_SIR_max,” respectively, as can a graphical representation of these models with function “plot_predict.” For SIR models, only simulations where all individuals had been infected at some point in the simulation were considered sufficient. This resulted in exclusion of 26.9% of simulations in which the disease failed to infect every individual. The purpose of this screening was to ensure that a single continuous metric, outbreak duration, could be used to compare all simulations.

To determine under which conditions our method would be most useful, regression models were calculated with raw network size as the response and outbreak duration as the predictor. The association between raw network size and outbreak duration was exponential rather than linear, as would be expected from an exponential growth system like disease transmission in SI models (27). To determine the area of the graphs where we could reliably predict network size from outbreak duration, we used piecewise OLS regressions to predict two separate relationships between outbreak. We did not transform these data at this point, because by splitting the relationship into two separate regressions with piecewise regression, this approach allowed us to identify portions of the graph where prediction could be made appropriately. In the first portion, duration outbreak would show a relatively shallow relationship with network size, making prediction reasonable. But in the second, much steeper portion, relatively small increases in outbreak duration would show much larger increases in predicted network size, making prediction tenuous. We estimated piecewise regression models in R with package segmented (28) to determine where the breakpoint between the two portions of the graph would be; this method optimizes the linear fit of each portion by randomly varying the breakpoint until the best split is achieved. This can be replicated in enss with function “breakpoint_max.” We also simulated the simpler SI models with varying values of β to determine if raising or lowering this parameter had any effect on the breakpoint in these piecewise regressions. Such a result would indicate that altering β would allow for better or worse predictions of large network sizes from longer outbreak durations.

In addition to considering piecewise regression models, we separately ran regression models with log-transformed network sizes to achieve a linear fit. For each set of 1,000 iterations of disease simulation on a given network, outbreak durations were quite variable. Thus, we used reduced major axis (RMA), estimates of model II regressions to control for the uncertainty in outbreak duration in addition to that in network size, calculated in R with package lmodel2 (29). RMA estimates consider the variation in both the independent and dependent variable when fitting regression models rather than, as in OLS models, only considering variation in the dependent variable. RMA provided the most suitable control for estimating how variation in outbreak duration would affect our predictions of fixed network sizes.

We then exponential transformed the output of these equations to back-transform for the log-transformation. These exponential-transformed equations formed the basis for calculating “effective” network sizes from outbreak durations of diseases simulated on observed networks. Back-transformations from log-transformed data introduce bias into predicted values because of the difference between errors in log-transformed variables and their untransformed counterparts (30, 31). We considered accounting for this bias by using the “consistent I estimator” from Hayes and Shonkwiler (30), and compared this approach to our own method of calculating network size from the uncorrected RMA models; the equation for the consistent I estimator is:

$$y={\text{e}}^{\text{ln}\left(a\right)+b\text{ln}\left(x\right)+\left(\frac{s^{2}}{2}\right)}$$

where *a* is the intercept, *b* is the slope, *x* is the independent variable, and *s*^2^ is the mean squared error for the model. Because mean squared error is constant within each model, such a correction would create a consistent upward shift in all estimates of network size by a value of *s*^2^/2; this would not have any impact on further linear models’ slope coefficients, and so uncorrected RMA model back-transformations were chosen for simplicity of interpretation throughout the main text. Back-transformed predictions can be obtained in enss with function “estimate_backtrans_ens.” Comparisons of observed versus effective outbreak duration distributions are given in Figures S2 and S3 in Supplementary Material.

### Accuracy, Precision of Predicting ENS From Randomly Structured Graphs

To investigate the second aim, we generated large sets of Erdős-Rényi (E-R) graphs (Figure 1C) for predetermined group sizes and predetermined density of ties present; to reduce variability, these were used as set numbers of ties, rather than probability that ties would be present between two given nodes, as is more typical in density-determined E-R graphs in R with package igraph (24). Random graphs were used as the baseline in this case because they represented the only source from which we could obtain a large enough sample size to validate our methods. Group sizes for these were kept smaller than the maximally complete networks to allow for direct comparison of outbreak duration distributions, and they are in good agreement with the observed network sizes of primates ranging from 4 to 35 typically (32). Tie proportions were kept relatively low to increase differentiation from maximally complete networks. We sampled blocks of 111 networks for each combination of group size (*n* = 10, 30, and 50) and tie proportion (15, 25, and 35% of possible ties), generating 999 total random networks. To ensure that disease simulations could reach full saturation and (for SIR) subsequent extinction, we screened each randomly generated network to ensure that all nodes were part of a single, connected network. This method can be reproduced using function “gen_erg” in package enss.

We then simulated the same SI and SIR models (as discussed in Section “Simulation and Regression of Disease Transmission on Maximally Complete Networks”) over 1,000 iterations on each of our 999 randomly generated models, recording outbreak durations of the models (again with enss functions “clust_sim_SI” and “clust_sim_SIR,” respectively). Because all outbreak durations for random networks of size *N* are expected to be greater than those of the idealized network of size *N*, these simulations were conducted to determine the scale of increase in outbreak durations and consequently in ENS. The mean of outbreak durations for a given random network with a given epidemiological model were used as the predictor variable in the RMA regression equations described in Section “Simulation and Regression of Disease Transmission on Maximally Complete Networks.” Only simulations which reached saturation were analyzed here, and so some runs of the SIR simulations were removed due to stochastic extinction events. This reduced the sample size of analyzed simulation runs and may have biased our results for SIR comparisons. These values were then exponential-transformed and rounded to the nearest integer to arrive at a directly comparable ENS for each random network (using enss function “estimate_backtrans_ens”). Thus, ENS were calculated twice for each random network; once for SI models and once for SIR models.

To gauge the accuracy and precision of our methods, we compared each distribution of outbreak durations on a given E-R network (hereafter, called the “observed network”) to that of the original outbreak durations on the maximally complete network of the same size as the predicted ENS of the observed network (hereafter, “effective network”). We compared these distributions graphically (with enss functions “plot_compare_SI” and “plot_compare_SIR”) and statistically (with enss functions “compare_SI_erg_ens” and “compare_SIR_erg_ens”). For accuracy, we compared the observed and effective network distributions in means of outbreak durations, with more similar means indicating that simulating disease spread on effective networks is more accurately capturing expected spread on the observed network. For precision, we compared the observed and effective network distributions in SDs of outbreak durations, with more similar SDs indicating that the precision of simulating disease spread on effective networks is similar to what would be obtained on the actual networks. We statistically compared the distributions of outbreak durations between observed and effective network simulations with Kolmogorov–Smirnov tests in R with package dgof (33). Significance on these tests indicates that the two distributions likely did not come from the same original distribution.

### Associations Between ENS and Other Metrics

As one example application of our methods, we used our predictive models to estimate ENS of primate social networks that had been recorded in the literature (e.g., Figure 1D). These networks mainly consisted of the dataset of weighted sociomatrices collected by Griffin and Nunn (26), supplemented with more recent publications. Batch importing of empirical social networks was accomplished in enss using function “import_emp.” A full listing of the sources for each of these networks, as well as the species and interaction type to which each corresponds, is provided in Table 1. ENS were again calculated by simulating SI and SIR models and then inputting the resulting outbreak duration means into the equations described in Section “Simulation and Regression of Disease Transmission on Maximally Complete Networks.” For each of the empirical social networks, we then calculated weighted and unweighted versions of five common network metrics leading eigenvector modularity, which is a measure of how subdivided a network is into cliques, with higher values indicating more extreme subdivision, was calculated using function leading.eigenvector.community from package igraph (24). Mean distance, or the average of the shortest paths between each combination of two nodes, was calculated using function distance_w from package tnet (34); greater distances between nodes indicate that information will take longer to spread across the network. A related metric, diameter, measures the longest of these shortest paths across the entire network; it was calculated using function diameter from package igraph (24). Clustering coefficient, a measure of complete connectedness among triplets of nodes which have at least two connections among them, was calculated using function clustering_w from package tnet (34); higher clustering coefficients indicate that if three nodes are connected by at least two connections, they likely also include the third connection. Eigenvector centralization measures the skewness in the centrality, or connectedness of each node within the network, with higher values indicating greater skew from a uniform distribution of centralities; this was calculated using function evcent from package igraph (24). These metrics can be calculated for any set of networks using the “calculate_metrics” function in enss, which simply automates the calculations performed by functions provided in packages igraph (24) and tnet (34). Then, we compared models with all combinations of these metrics as predictors of ENS in a model comparison framework with AICc as the model selection criterion, using a cutoff of two AICc units for preferring a model over other models. AICc values were calculated in R with package MuMIn (35) and can be calculated in batch form with the enss function “AICc_ens_metrics.”

**Table 1:** Raw and ENSs of primate species included in the established metric comparison models, as well as source information for each of the networks.

Species	Group size	ENS SI	ENS SIR	Weighted ENS SI	Weighted ENS SIR	Group status	Interaction class	Source
*Alouatta caraya*	5	7	8	9	7	Captive	Grooming	(36)
*Ateles geoffroyi*	15	36	22	75	23	Free-ranging	Grooming	(37)
*Cebus apella*	12	20	18	43	18	Wild	Grooming	(38)
*Cebus capucinus*	6	9	10	9	9	Wild	Grooming	(39)
*Cercopithecus aethiops*	8	11	13	15	12	Wild	Grooming	(40)
*Cercopithecus mitis*	16	43	21	57	26	Wild	Grooming	(41)
*Colobus guereza*	8	13	13	43	13	Wild	Grooming	(42)
*Eulemur fulvus*	11	16	16	20	15	Free-ranging	Proximity	(43)
*Lemur catta*	12	16	17	20	16	Wild	Proximity	(44)
*Macaca arctoides*	19	31	26	53	26	Captive	Grooming	(45)
*Macaca assamensis*	19	36	26	79	28	Wild	Grooming	(46)
*Macaca fascicularis*	10	20	15	70	17	Captive	Grooming	(47)
*Macaca mulatta*	28	34	35	37	30	Captive	Proximity	(48)
*Macaca radiata*	16	25	22	32	22	Wild	Grooming	(49)
*Miopithecus talapoin*	8	11	13	16	12	Captive	Grooming	(50)
*Pan troglodytes*	7	10	11	12	9	Wild	Grooming	(10)
*Papio ursinus*	14	24	21	27	18	Wild	Grooming	(51)
*Saguinus fuscicollis*	7	10	12	16	11	Captive	Grooming	(52)
*Saguinus mystax*	6	9	10	10	9	Wild	Grooming	(53)
*Theropithecus gelada*	7	15	12	16	10	Captive	Sociopositive	(54)

*“Network size” is the count of nodes in the observed primate network. “ENS” indicates effective network size, with “SI” or “SIR” indicating the type of transmission model used for estimating ENS, and “weighted” indicating that tie weights were also included in simulations for estimating ENS. In some cases, weighted ENS measures were very different from their unweighted counterparts, indicating a strong effect of adding in tie weight information. In other cases, SI and SIR ENS estimates varied widely within species; these typically indicate an effect of removing non-total transmission simulations from SIR models, lowering ENS*.

*SI, susceptible-infected; SIR, susceptible-infected-recovered*.

## Results

**Figure 2:**
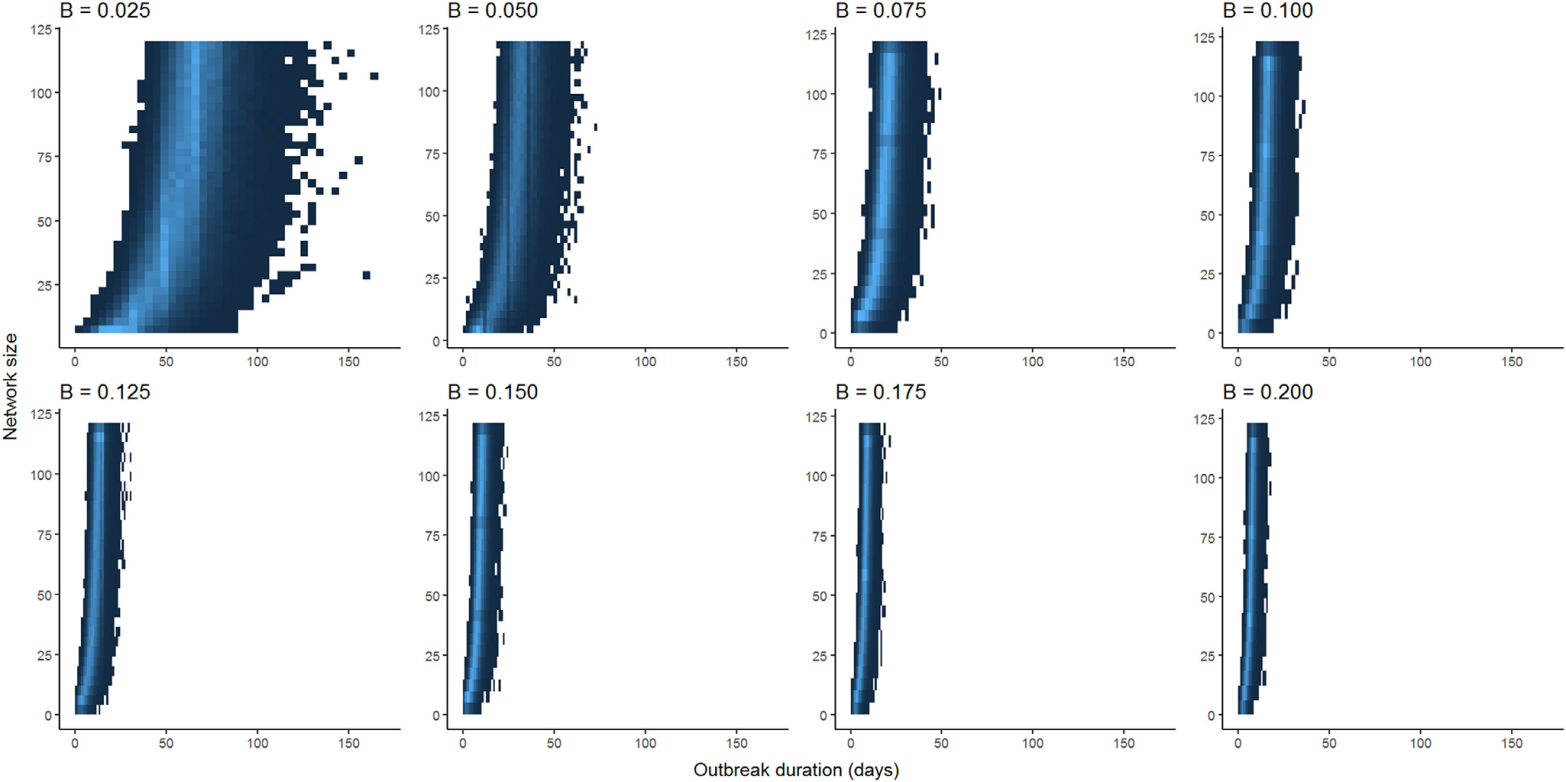
Comparison between distributions of outbreak durations for susceptible-infected simulations with varying values for β. Lower values for β have larger ranges of outbreak durations, but the shapes of curves are qualitatively similar when scaled to the maximum outbreak duration for a given value of β.

**Figure 3:**
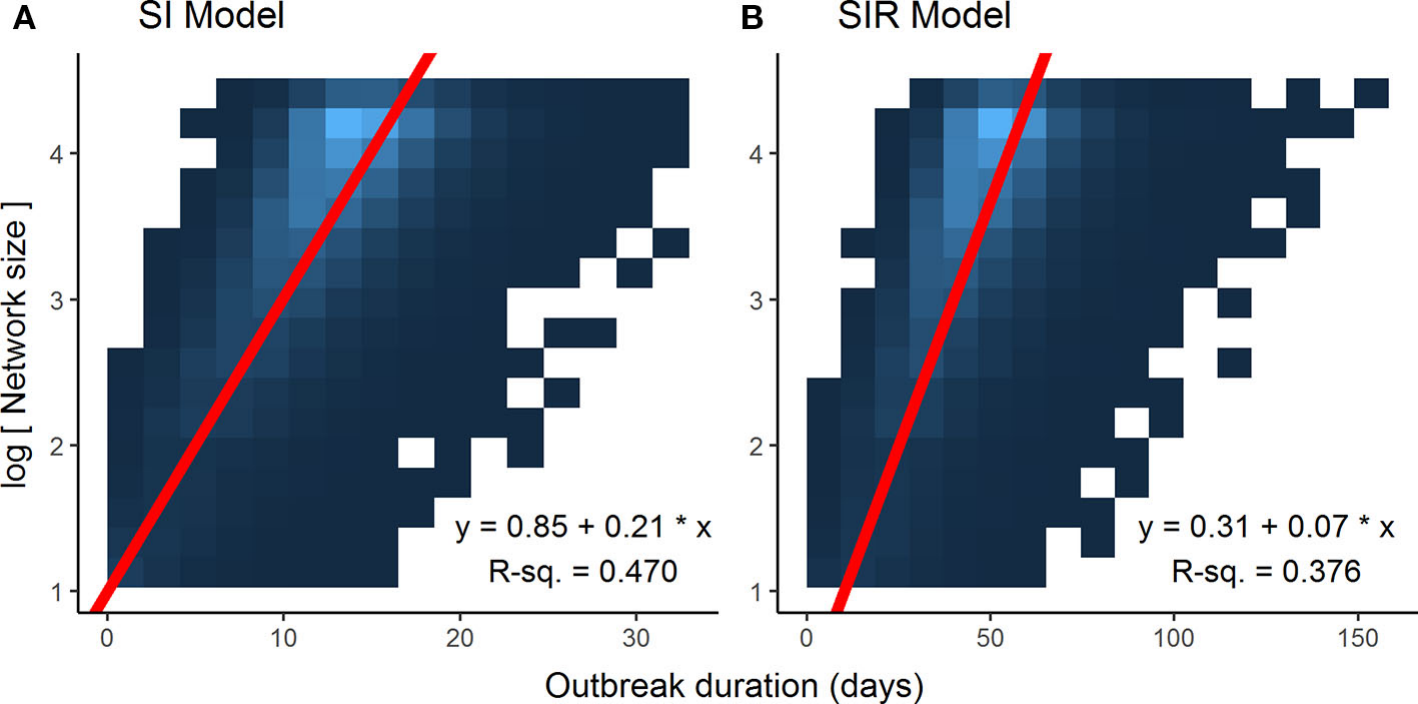
Associations between log-transformed network size and outbreak duration for different disease models. Data points for each graph, limited to networks of 80 nodes or less (*n* = 78,000), were too dense to make scatterplot representations intelligible, thus heatmaps were used to illustrate the results, with lighter colors of blue representing a higher density of data points. Log-transforming network size makes for a linear relationship, and reduced major axis model 2 regression lines, represented in red, account best for the joint variation in the *x* and *y* axes. **(A)** Susceptible-infected (SI) model. **(B)** Susceptible-infected-recovered (SIR) model.

Optimization of piecewise regression models estimated a break at a network size of 80 nodes, indicating that predictions of ENS above 80 individuals would be considerably less reliable than those of 80 or below. Furthermore, altering the values for β had no effect on the breakpoints, although as would be expected, the ranges of outbreak durations were inversely related to the value for β (Figure 2). All piecewise regressions revealed breaks at between 79.45 and 81.75 nodes. RMA model II regressions of log-transformed maximally complete network size versus outbreak duration for SI and SIR models fit relatively well, with *R*^2^ of 0.470 and 0.376, respectively (Figure 3). The regression equations, listed in Figures 3A,B, were then used to calculate ENS. Alternative model results, with ties sampled regularly rather than randomly, showed similar results for SIR models, but tended to oversample ties in large networks for SI models, leading to unreasonably short outbreak durations in these networks (Figure S1 in Supplementary Material).

**Figure 4:**
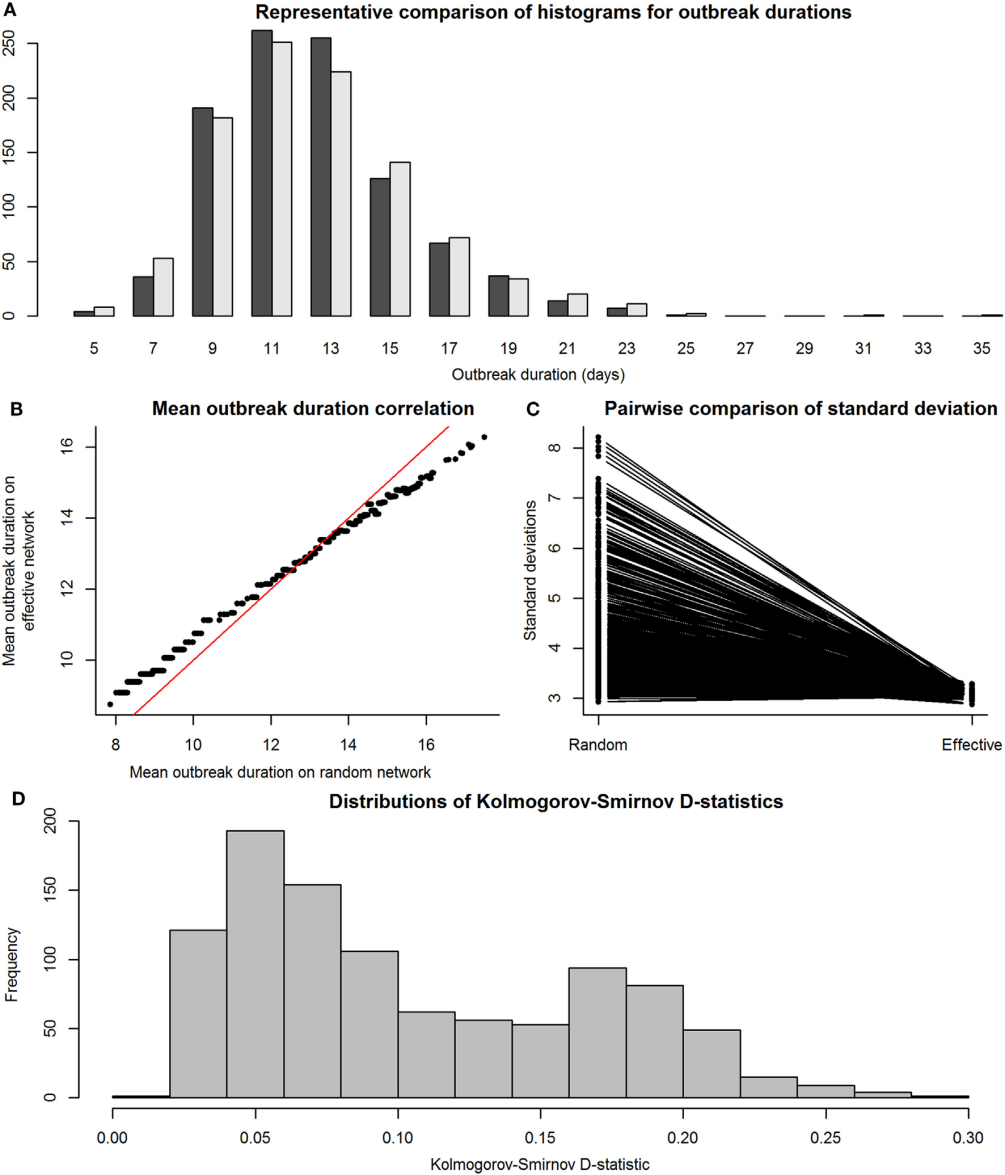
Comparison between distributions of outbreak durations for susceptible-infected simulations on observed and effective network. Throughout the figure, the term “observed” refers to results from simulations on Erdős-Rényi graphs, and “effective” refers to results from simulations on reduced major axis-predicted equivalent maximally complete networks. Network sizes are limited to a maximum of 80 individuals, as this was the condition under which we were reasonably confident in our results. Panel **(A)**, a histogram with a representative pair of observed (dark gray) and effective (light gray) distributions of outbreak durations plotted together for viewing overlaps, shows that the distributions, compared on a pairwise scale had a considerable amount of overlap. Panel **(B)** shows means of outbreak durations from observed networks plotted against those from their predicted effective networks; red line indicates 1:1 equivalence, at which effective means match observed means. Panel **(C)** shows a paired line plot of SDs in outbreak durations for simulations on observed and effective networks; observed networks showed higher SDs than their paired effective networks. Panel **(D)** shows a histogram of Kolmogorov–Smirnov D-statistics for pairwise statistical comparisons between observed and effective network outbreak durations, with values above 0.60 indicating significantly different distributions.

We then compared the distributions of E-R graph (observed) outbreak durations to those of their equivalent maximally complete (effective) network’s outbreak durations to assess accuracy and precision. This was done to determine whether disease outbreaks on observed networks were accurate, or similar to those on maximally complete networks, in terms of the distributions of the outbreak durations from simulations on effective and observed networks. Figure 4 shows the results of the SI model comparisons. Accuracy of our RMA predictive model was high, with means similar between observed and effective network outbreak durations (Figure 4B), but outbreak durations from observed network simulations showed higher SDs than those from effective networks (Figure 4C). Kolmogorov–Smirnov tests show that these two sets of distributions were often significantly different, with a critical value for the D-statistic at 0.60 (Figure 4D). However, this method is extremely sensitive to small changes in distributions and may not be best suited for determining similarity between the observed and effective network outbreak duration distributions.

**Figure 5:**
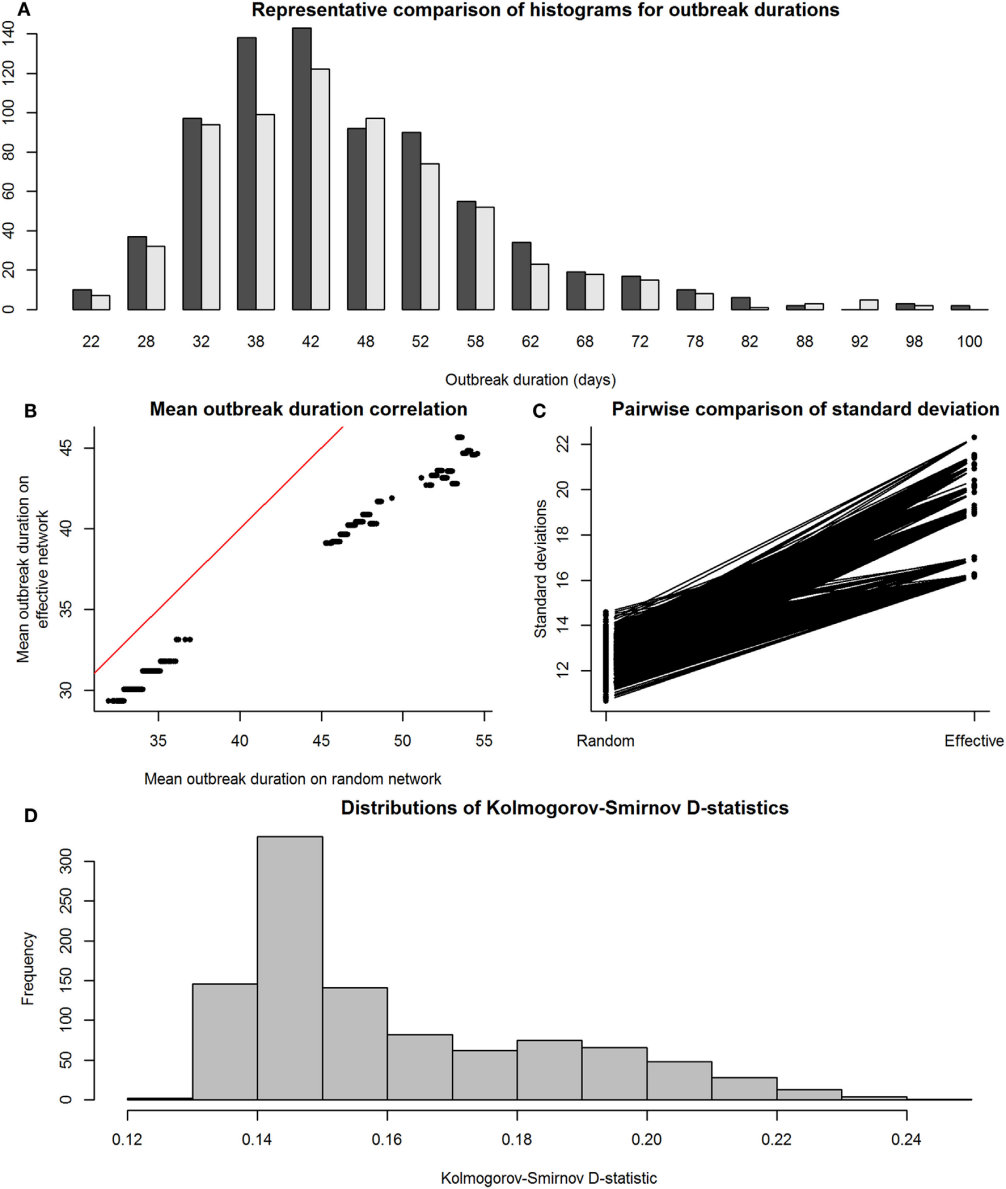
Comparison between distributions of outbreak durations for susceptible-infected-recovered simulations on observed and effective network. Again, the term “observed” refers to results from simulations on Erdős-Rényi graphs, and “effective” refers to results from simulations on reduced major axis-predicted equivalent maximally complete networks. Network sizes are also limited to a maximum of 80 individuals, as this was the condition under which we were reasonably confident in our results. Panel **(A)**, a histogram with a representative pair of observed (dark gray) and effective (light gray) distributions of outbreak durations plotted together for viewing overlaps, shows that the distributions, compared on a pairwise scale had a considerable amount of overlap. Panel **(B)** shows means of outbreak durations from observed networks plotted against those from their predicted effective networks; red line indicates 1:1 equivalence, at which effective means match observed means. Panel **(C)** shows a paired line plot of SDs in outbreak durations for simulations on observed and effective networks; observed networks showed higher SDs than their paired effective networks. Panel **(D)** shows a histogram of Kolmogorov–Smirnov D-statistics for pairwise statistical comparisons between observed and effective network outbreak durations, with values above 0.60 indicating significantly different distributions.

Figure 5 shows the results of the SIR model comparisons between effective and observed network simulations. Again, similarity between mean values of outbreak durations for simulations on effective and observed networks (i.e., accuracy) was high (Figure 5B), but outbreak durations from observed network simulations actually showed lower SDs than those from effective networks (Figure 5C); this was likely due to the exclusion of simulations where the disease went extinct, which would have drastically reduced the variance of results. Kolmogorov–Smirnov tests show that these two sets of distributions were often significantly different, again with a critical value for the D-statistic at 0.60 (Figure 4D).

In our model selection framework comparing unweighted ENS to other established unweighted network metrics, the best fitting model for SI ENS included positive associations with raw group size (*b* = 1.13), mean distance (*b* = 116.93), and clustering coefficient (*b* = 66.71), as well as a negative association with eigenvector centralization (*b* = −107.31); the model had an adjusted *R*^2^ of 0.971. There were four best fitting models for SIR ENS within two units of the minimum AICc value, and thus each of the following models were tied for best fit: SIR best fit #1 included positive associations with raw group size (*b* = 1.15), clustering coefficient (*b* = 3.44), and eigenvector centralization (*b* = 17.61); the model had an adjusted *R*^2^ of 0.993. SIR best fit #2 included positive associations with raw group size (*b* = 1.16) and eigenvector centrality (*b* = 20.27), as well as a negative association with mean distance (*b* = −4.25); the model had an adjusted *R*^2^ of 0.993. SIR best fit #3 included positive associations with raw group size (*b* = 1.17) and eigenvector centralization (*b* = 17.23), as well as a negative association with leading eigenvector modularity (*b* = −13.35); the model had an adjusted *R*^2^ of 0.993. SIR best fit #4 included positive associations with raw group size (*b* = 1.15) and eigenvector centralization (*b* = 5.69); the model had an adjusted *R*^2^ of 0.992.

Meanwhile, for the weighted models, the best fit for weighted SI ENS included positive associations with raw group size (*b* = 2.40) and mean weighted distance (*b* = 51.26), as well as a negative association with weighted diameter (*b* = −15.15); the model had an adjusted *R*^2^ of 0.706. Again, there were four best fitting models for weighted SIR ENS within two units of the minimum AICc value, and thus each of the following models were tied for best fit: weighted SIR best fit #1 included positive associations with raw group size (*b* = 1.14), leading eigenvector modularity (*b* = 15.88), and eigenvector centralization (*b* = 4.29); the model had an adjusted *R*^2^ of 0.949. Weighted SIR best fit #2 included positive associations with raw group size (*b* = 1.16) and mean weighted distance (*b* = 5.30), as well as a negative association with weighted diameter (*b* = −1.84); the model had an adjusted *R*^2^ of 0.949. Weighted SIR best fit #3 included positive associations with raw group size (*b* = 1.13), mean weighted distance (*b* = 1.83), and leading eigenvector modularity (*b* = 14.44); the model had an adjusted *R*^2^ of 0.950. Weighted SIR best fit #4 included positive associations with raw group size (*b* = 1.10) and leading eigenvector modularity (*b* = 18.15); the model had an adjusted *R*^2^ of 0.948.

## Discussion

These results demonstrate the potential for using ENS to compare infectious disease risk across groups of different sizes, including potentially for understanding disease transmission across a mosaic of many loosely connected groups within a larger meta-population structure, as well as for simplifying entire meta-populations to a single ENS. Previous studies have applied similar network-level metrics, like centrality and modularity, to the study of disease transmission through contact, grooming, and sociopositive networks in both wild and captive populations (32, 55–59). But nearly all of these measures capture only one aspect of networks, and they require this aspect to be considered in isolation from other important information about the network, specifically, its size. This issue is especially problematic for some metrics like modularity, whose value is mathematically positively associated with network size (22, 60). When compared to established network metrics, our single metric of ENS was best predicted by a combination of group size plus at least two other metrics. Thus, our measure of ENS provides a metric for disease transmissibility among individuals in a group that also accounts for the size of the group from which it was estimated. This differs from the previously mentioned approach by Caillaud et al. (12), which focused on understanding sub-group heterogeneity of meta-populations in light of epidemic thresholds. Specifically, our approach uses network structure and group size to predict how quickly a disease can be transmitted and maintained by individuals in a population.

Many more established network metrics covaried consistently with our measures of ENS, although there were differences most noticeably between transmission modes. For both SI and SIR ENS, the raw, original group size (number of nodes in the observed network) covaried strongly and positively with ENS, supporting the claim that ENS presents a novel, “size standardized” network metric. In addition, for SI models, both weighted and unweighted, mean distance was positively associated with ENS, perhaps indicating that SI models function through a simple diffusion process, where distance traveled is the best indicator of disease spread time. On the other hand, for SIR models, again both weighted and unweighted, network metrics like centralization and modularity, which generally indicate the skewness of tie distributions, showed generally positive associations with ENS. These relationships may point more toward the importance of skewness of connections in impeding or bottlenecking the spread of diseases specifically for SIR transmission models.

Of course, social networks can be represented in many ways, and our approach still simplifies networks considerably from their real-world manifestations. First, nearly all social ties in the real world vary in intensity (i.e., the networks are weighted), yet we conducted most of our tests using unweighted networks. The unweighted networks were used as a less “noisy” test of our methods. We did, however, also test for associations between ENS and other network metrics using weighted primate networks, which generally showed weaker effects compared with using unweighted ENS, likely due to the increased variation introduced by tie weights. Additional sources of variability are also worth considering. For example, individuals may vary in traits that make them more or less susceptible to a disease or to transmitting it, including trade-offs between reproductive status, dominance, and immune system, as well as age-related effects on immune function (59). Networks may also vary in their structure across time, adding yet another variable that complicates analyses (58, 61–63). However, the majority of research focuses on the importance of structural aspects of static networks for predicting and mitigating disease transmission, as this allows for more straightforward interpretation and comparison among different populations (64–66).

Additional applications of the method may open a variety of new routes for wildlife management and infection control. ENS could be used in disease outbreak risk assessments for wild or captive populations with known social networks. In addition, meta-populations of groups with known social networks could be simplified to their respective ENS to make prediction of future outbreaks easier in the future. Groups of sufficient size or structure could be targeted for vaccination campaigns in the wild or in captivity. In addition to comparing groups in a meta-population to one another, ENS could be used as a rough heuristic at a larger scale, reducing entire meta-populations to a single ENS. Finally, if further work is conducted to develop our method into a mathematical one rather than a simulation-based one, this approach could be applied to policy and management applications where simulation modeling is prohibitively time-consuming.

Although this study has only focused on simulation-based solutions for determining ENS, mathematical solutions for determining ENS should be investigated to obtain more succinct and resource-efficient calculations. One such approach for these mathematical solutions was shown by Caillaud et al. (12), but mathematicians and theoreticians interested in the effects of group size on disease transmission could still significantly further such research. In addition to this, the number of studies that have published social network structures is still small. For this reason, we encourage scientists researching social interaction to publish network information on species for which they already have data and to begin more studies of social network analysis in primate groups.

## Author Contributions

CM and CN designed the research and wrote the manuscript. CM developed R code and analyzed data.

## Conflict of Interest Statement

The authors declare that the research was conducted in the absence of any commercial or financial relationships that could be construed as a potential conflict of interest.
