# Supplementary material for: Effective Network Size Predicted From Simulations of Pathogen Outbreaks Through Social Networks Provides a Novel Measure of Structure-Standardized Group Size
Source: Front Vet Sci. 2018 May 3;5:71. doi: 10.3389/fvets.2018.00071 (PMC5943561; doi:10.3389/fvets.2018.00071)
Supplement: Supplementary file 1 [file data_sheet_1.PDF]

Supplementary Material

Effective Network Size Predicted from Simulations of Pathogen Outbreaks through Social Networks Provides a Novel Measure of Structure-standardized Group Size

Collin M. McCabe*, Charles L. Nunn

*** Correspondence:** Collin M. McCabe: collin.michael.mccabe@gmail.com

1 Supplementary Data

By simulating disease spread on maximally complete networks, we inevitably encountered issues of the number of ties in a network not growing proportionally to the number of nodes. This relationship is due to the exponential relationship between network size and the maximum number of ties in that network, as shown in Supplementary Equation 1:

$$\boldsymbol{Ties\ in\ Network\ of\ size\ N = \frac{N(N-1)}{2} = \frac{N^2 - N}{2}}$$

By simulating the spread of disease on a network of given size N, if we wish to represent each tie once in a day of simulations, the number of ties sampled will be exponentially greater than the number of nodes (for any network larger than 2, which all of our networks were). This leads to unreasonably rapid disease outbreaks at large network sizes. For this reason, a per capita interaction rate, such as 3 interactions per node, was chosen, because scaling interactions per day by the size of a network resulted in a total number of ties selected per day that scaled linearly with network size, as shown in Supplementary Equation 2:

$$\boldsymbol{\frac{3N}{N(N-1)/2} = \frac{6}{N-1}}$$

2 Supplementary Figures and Tables

2.1 Supplementary Figures

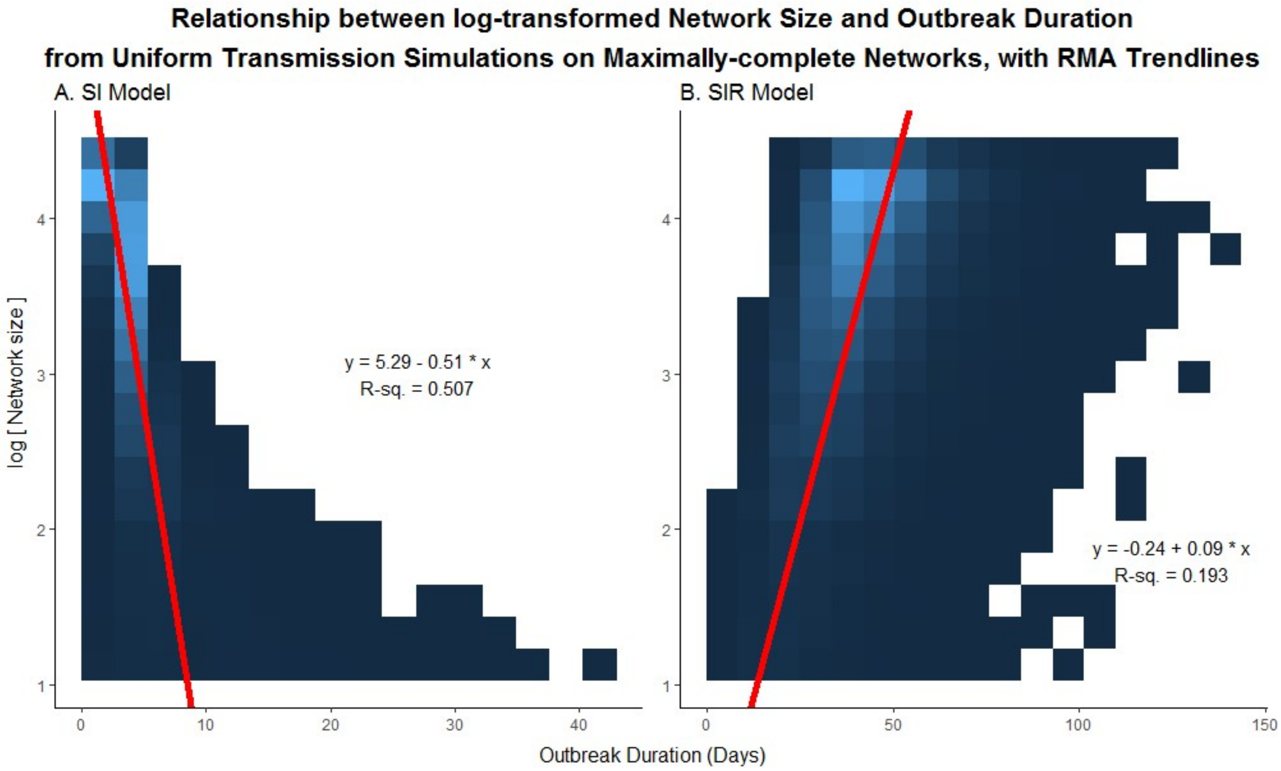

Supplementary Figure 1. Associations between log-transformed network size and outbreak duration for different disease models using alternative models with uniform tie selection per day rather than random tie selection using per capita interaction rates per day. Data points for each graph, limited to networks of 80 nodes or less (n=77,000), were too dense to make scatterplot representations intelligible, thus heatmaps were used to illustrate the results, with lighter colors of blue representing a higher density of data points. Log-transforming network size makes for a linear relationship, and reduced major axis (RMA) model 2 regression lines, represented in red, account best for the joint variation in the x and y axes. Both SI and SIR models show a much steeper increase in outbreak duration as network size increases than do the per capita interaction rate models, but the relationship is most apparent in SI models, where recovery does not counteract the spread of disease. Given the exponential relationship between network size and the maximum number of possible ties present in that network [Supplementary Equation 1], there are more and more possible routes for disease transmission through a maximally connected network as the number of nodes increases, causing a seemingly negative relationship between network size and outbreak duration. For this reason, per capita interaction rates for networks were chosen as a more representative transmission model for our methods.

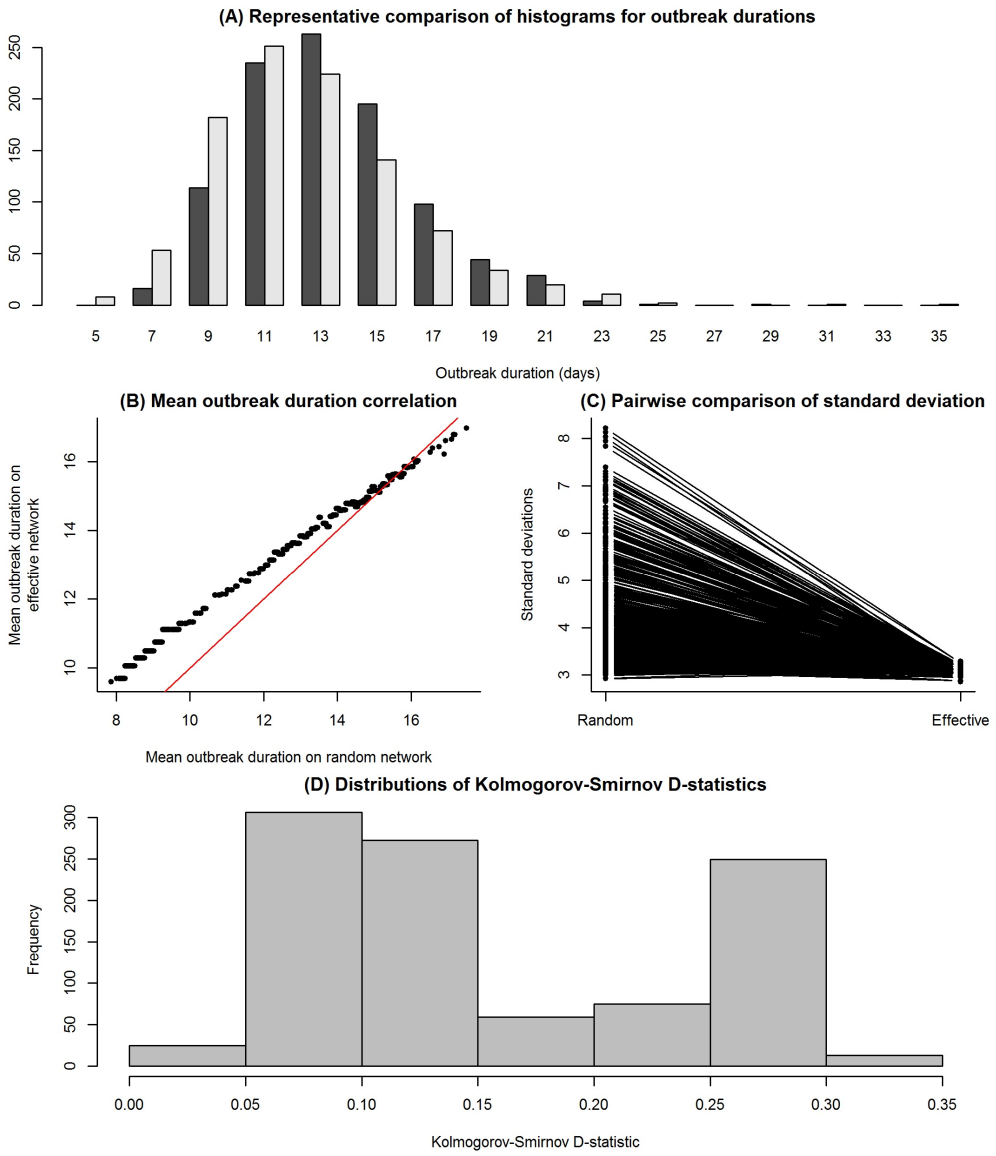

Supplementary Figure 2. Comparison between distributions of outbreak durations for SI simulations on observed and effective network, where effective network size estimates have been back-transformed by the consistent I estimator (24). Throughout the figure, the term "observed" refers to results from simulations on E-R graphs, and "effective" refers to results from simulations on RMA-predicted equivalent maximally-complete networks. Network sizes are limited to a maximum of 80 individuals, as this was the condition under which we were reasonably confident in our results. Panel A, a histogram with a representative pair of observed (dark gray) and effective (light gray) distributions of outbreak durations plotted together for viewing overlaps, shows that the distributions, compared on a pairwise scale had a considerable amount of overlap. Panel B shows means of outbreak durations from observed networks plotted against those from their predicted effective networks; red line indicates 1:1 equivalence, at which effective means match observed means. Panel C shows a paired line plot of standard deviations in outbreak durations for simulations on observed and effective networks; observed networks showed higher standard deviations than their paired effective networks. Panel D shows a histogram of Kolmogorov-Smirnov D-statistics for pairwise statistical comparisons between observed and effective network outbreak durations, with values above 0.60 indicating significantly different distributions.

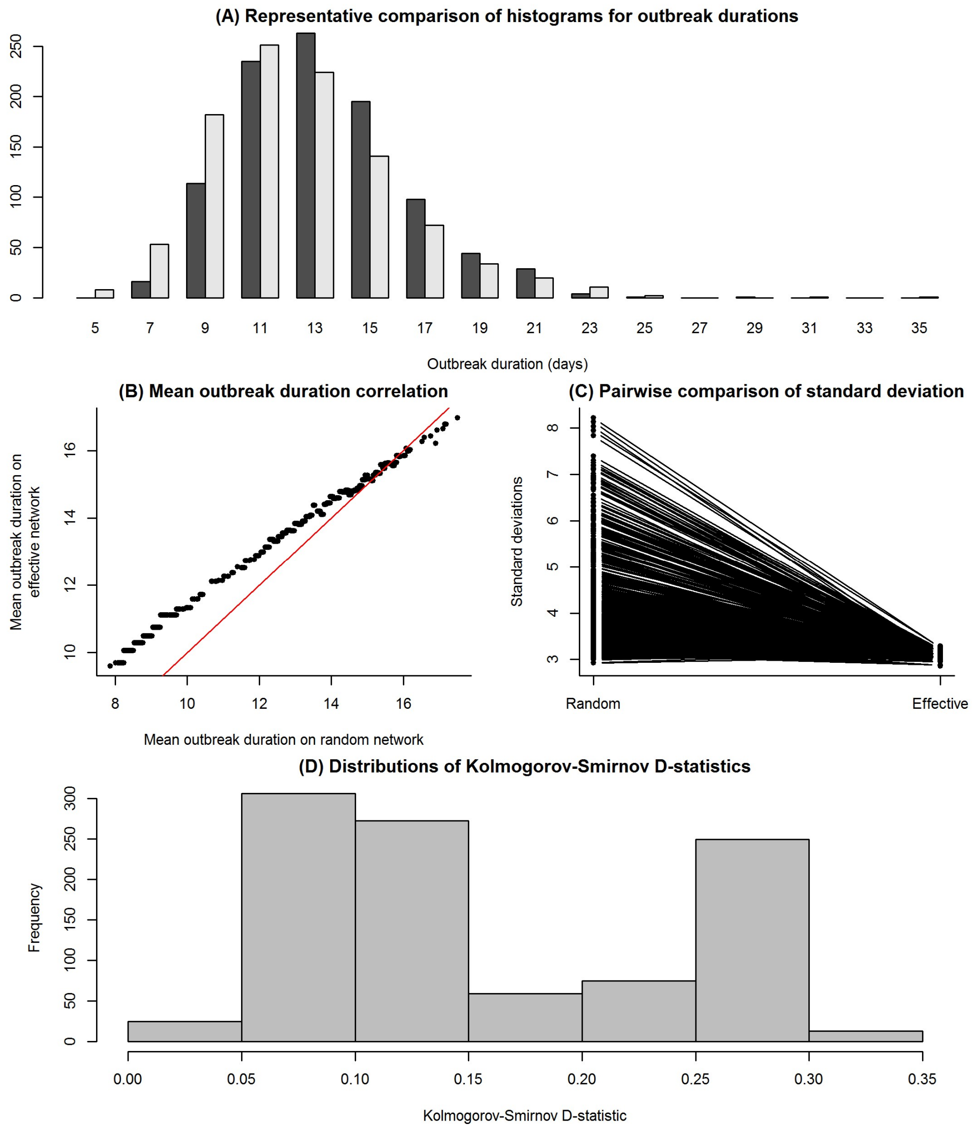

Supplementary Figure 3. Comparison between distributions of outbreak durations for SIR simulations on observed and effective networks, where effective network size estimates have been back-transformed by the consistent I estimator (24). Again, the term "observed" refers to results from simulations on E-R graphs, and "effective" refers to results from simulations on RMA-predicted equivalent maximally-complete networks. Network sizes are also limited to a maximum of 80 individuals, as this was the condition under which we were reasonably confident in our results. Panel A, a histogram with a representative pair of observed (dark gray) and effective (light gray) distributions of outbreak durations plotted together for viewing overlaps, shows that the distributions, compared on a pairwise scale had a considerable amount of overlap. Panel B shows means of outbreak durations from observed networks plotted against those from their predicted effective networks; red line indicates 1:1 equivalence, at which effective means match observed means. Panel C shows a paired line plot of standard deviations in outbreak durations for simulations on observed and effective networks; observed networks showed higher standard deviations than their paired effective networks. Panel D shows a histogram of Kolmogorov-Smirnov D-statistics for pairwise statistical comparisons between observed and effective network outbreak durations, with values above 0.60 indicating significantly different distributions.

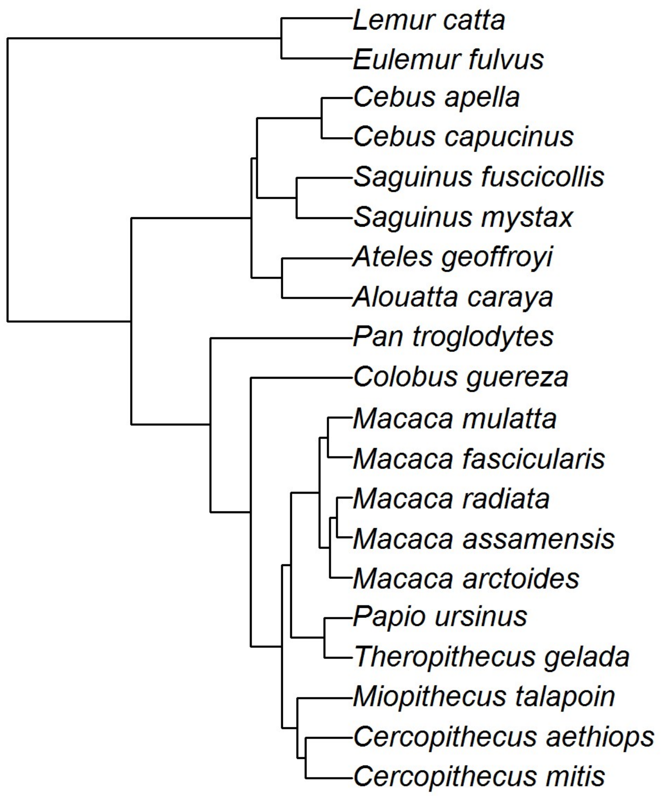

Supplementary Figure 4. Phylogenetic tree of primate species for which we tested hypotheses about associations between group size and parasite richness. Tree shows poor coverage of certain taxa of primates, such as strepsirrhines, for which only 2 species, Lemur catta and Eulemur fulvus, had published social network data.

2.2 Supplementary Tables

Supplementary Table 1. Richness model comparison between effective network sizes, observed raw network size, geographic range size, and body mass for 22 primate species. Reported are slope estimates, p-values, and AICc values for each model. Models are indicated by the independent variable in rows ($N_{en,SI}$ = effective network size predicted from SI equation; $N_{en,SIR}$ = effective network size predicted from SIR equation; N_{obs} = observed raw network size; GR = geographic range; BM = body mass) and the dependent variable, which specific measure of parasite richness was used, in columns.

	**Parasite Species Richness**				
	Total	*Close-transmitted*	*Helminth*	*Protozoa*	*Virus*
$N_{en,SI}$	β = -0.238<br>p = 0.253<br>AICc = 55.6	β = -0.105<br>p = 0.653<br>AICc = 51.2	β = -0.241<br>p = 0.288<br>AICc = 49.4	β = -0.174<br>p = 0.459<br>AICc = 47.6	β = -0.198<br>p = 0.422<br>AICc = 33.8
$N_{en,SIR}$	β = -0.270<br>p = 0.201<br>AICc = 55.2	β = -0.151<br>p = 0.544<br>AICc = 51.0	β = -0.292<br>p = 0.215<br>AICc = 48.9	β = -0.356<br>p = 0.133<br>AICc = 45.4	β = 0.071<br>p = 0.784<br>AICc = 34.5
N_{obs}	β = -0.256<br>p = 0.227<br>AICc = 55.4	β = -0.132<br>p = 0.603<br>AICc = 51.2	β = -0.285<br>p = 0.232<br>AICc = 49.0	β = -0.340<br>p = 0.157<br>AICc = 45.7	β = 0.069<br>p = 0.785<br>AICc = 34.5
GR	β = 0.188<br>p = 0.409<br>AICc = 56.4	β = 0.440<br>p = 0.171<br>AICc = 49.3	β = 0.047<br>p = 0.868<br>AICc = 50.8	β = 0.247<br>p = 0.389<br>AICc = 47.3	β = -0.040<br>p = 0.918<br>AICc = 34.6
BM	β = -0.124<br>p = 0.567<br>AICc = 56.8	β = 0.562<br>p = 0.020<br>AICc = 45.9	β = -0.196<br>p = 0.389<br>AICc = 49.9	β = 0.248<br>p = 0.353<br>AICc = 47.1	β = 0.287<br>p = 0.353<br>AICc = 33.5

Supplementary Table 2. Richness model comparison between effective network sizes, observed raw network size, geographic range size, and body mass for 20 primate species, using weighted models which sample ties in proportion to their weights. Reported are slope estimates, p-values, and AICc values for each model. Models are indicated by the independent variable in rows (Nen,SI = effective network size from SI equation; Nen,SIR = effective network size from SIR equation; Nobs = observed raw network size; GR = geographic range; BM = body mass) and the dependent variable, which specific measure of parasite richness was used, in columns.

	**Parasite Species Richness**				
	Total	*Close-transmitted*	*Helminth*	*Protozoa*	*Virus*
$N_{en,SI,w}$	β = -0.204<br>p = 0.328<br>AICc = 53.4	β = -0.257<br>p = 0.280<br>AICc = 47.7	β = -0.206<br>p = 0.361<br>AICc = 47.2	β = -0.255<br>p = 0.293<br>AICc = 44.6	β = -0.475<br>p = 0.062<br>AICc = 29.8
$N_{en,SIR,w}$	β = -0.227<br>p = 0.277<br>AICc = 53.1	β = -0.201<br>p = 0.423<br>AICc = 48.3	β = -0.235<br>p = 0.317<br>AICc = 47.0	β = -0.311<br>p = 0.201<br>AICc = 43.9	β = 0.239<br>p = 0.385<br>AICc = 33.7
N_{obs}	β = -0.256<br>p = 0.227<br>AICc = 52.7	β = -0.134<br>p = 0.599<br>AICc = 48.7	β = -0.287<br>p = 0.230<br>AICc = 46.4	β = -0.336<br>p = 0.168<br>AICc = 43.6	β = -0.069<br>p = 0.785<br>AICc = 34.5
GR	β = 0.376<br>p = 0.169<br>AICc = 52.2	β = 0.535<br>p = 0.117<br>AICc = 46.1	β = 0.253<br>p = 0.523<br>AICc = 47.7	β = 0.070<br>p = 0.879<br>AICc = 46.0	β = -0.040<br>p = 0.918<br>AICc = 34.6
BM	β = -0.110<br>p = 0.615<br>AICc = 54.2	β = 0.546<br>p = 0.025<br>AICc = 44.0	β = -0.182<br>p = 0.429<br>AICc = 47.4	β = 0.216<br>p = 0.432<br>AICc = 45.3	β = 0.287<br>p = 0.354<br>AICc = 33.5

3 Supplementary Analysis

3.1 Introduction

As an example of how to use effective network size, we also apply our new metric for representing disease transmission through networks to investigate the hypothesized links between group size and parasitism in primates (S3). Previous tests of this hypothesis have produced weak or non-significant results (S4-S6). We predicted that effective network size would be a better predictor of parasite richness than raw network size. We estimated the effective network size of primate social groups based on their social networks, and then test whether these estimates of effective network size explain variation in parasite richness across species. Because we do not have knowledge of the true underlying relationship between network characteristics and parasitism, this analysis is not a specific test of the method, but rather serves as an example application the method.

3.2 Methods

The consensus primate phylogeny used in our analysis [Supplementary Figure 4] was downloaded from 10kTrees, version 3 (S8), following the Corbet and Hill taxonomy (S9), and imported into R with package ape (S10). Parasite richness estimates considered were obtained from reports of parasitism in the scientific literature, and subsets including only socially-transmitted parasites and subsetted to helminths, protozoa, and viruses. Richness measurements were queried from the Global Mammal Parasite Database (S11) in December 2016 for each primate species. We tested the fit of the following linear model with PGLS analyses in R with package caper (S12):

$$\boldsymbol{Parasite\ Richness} = \boldsymbol{f}((\boldsymbol{Group\ size} \mid\mid \boldsymbol{Mass} \mid\mid \boldsymbol{Geographic\ range}) + \boldsymbol{Citation\ count})$$

Citation counts (the number of citations published up to December 2016 that were found in Web of Science for each primate species, controlling for taxonomic uncertainty) were included as a covariate in each model to control for sampling effort. We also included geographic range size estimates and mean body mass for each primate species from McCabe et al. (S1), as these have been previously supported in the literature as reliable predictors of parasite richness in primates (S1; S6). We conducted these analyses as a rough indicator of whether our small sample of primate species might impact the outcome of our tests; if we detected positive associations among these variables, but not among group size, then we could conclude that we had a representative sample of primate species.

Each measure of richness listed above was predicted to positively covary with group size. The measurements of group size tested were: observed (raw) sizes of primate networks, and effective network sizes from SI and SIR simulations, repeated for weighted and unweighted networks. All variables were log-transformed to meet assumptions of the PGLS analyses and z-transformed to facilitate comparison between different predictor variables (S13). Although some primates were represented multiple times in our dataset of published social networks, only one network per species ever met our condition of having an effective network size of less than cutoffs that were determined in the piecewise regression; thus, we did not need to account for any intraspecific variation. In total, 20 primate species were included in our analyses. Effective network sizes were predicted to be better estimators of parasite richness than the observed raw network sizes. We tested this prediction in a model comparison framework with AICc as the model selection criterion, using a cutoff of 2 AICc units for preferring a model over other models. AICc values were calculated in R with package MuMIn (S14).

3.3 Results

Results for PGLS analyses indicated, in general, no better performance of effective network size over raw network size in predicting parasite richness [Supplementary Table 1]. Similar results were found for weighted models, although AICc values for weighted models were lower across the board than were their equivalent unweighted ones for the same sample of species and richness [Supplementary Table 2]. Body size and geographic range were also not predictors of parasite richness across the board for the species included [Supplementary Table 1]. However, within these results, body mass was positively associated with close-contact transmitted parasite richness, and it had a lower AICc value than all other close-contact richness models.

3.4 Discussion

Unfortunately, as our PGLS results indicated, these novel measures of effective network size proved to be, in most cases, no better than raw group size at predicting parasite richness of a species. This comparative test was not intended to be a test of our novel method, but rather an application of the method to a standing issue in disease ecology. The fact that we found inconclusive results for this application does not invalidate our method, but rather, it could indicate any of a variety of other issues. In addition to the possibility that group size is not predictive of parasite richness (the hypothesis being tested), the estimates for parasite richness and primate social networks that we used in this study came from different sources, which might obscure any potential relationship between network structure and richness. Relationships between social networks and parasitism are more than likely dependent on the specific group being observed, and so ideally, measures of parasite richness and network structure should come from the same group. Additionally, the structure of a social network as well as the size of social groups are multifactorial phenomena, also affected by the availability of food, mates, and positions in dominance hierarchies (S2). Finally, most of our tests did not include parasites that exhibit exclusively social transmission. Empirical studies considering disease transmission on observed social networks have shown that relationships between network structure and parasite risk exist, but often when the parasite measures and network structures are taken from the same population (S15-S16), with a notable exception of network modularity showing negative associations with parasite richness in a comparative study of primates (S7).

However, network size and structure are not simply static traits of groups or species that affect exposure to pathogens, but mutable ones that also evolve in response to parasitism, competition within groups, and other selective pressures. We used phylogenetic models to test our predictions for group size and effective network size because many of these factors may covary with phylogeny, even if networks themselves do not. In response to increased pathogen pressures, a group may reduce its contact rate or restructure the contact network to avoid infected individuals and reduce transmission of the pathogen (S15), but this reduction in interaction must also be balanced by the innate drive of social animals to interact with one another (S17). Of course, other pressures (or more specifically, the relaxing of them), such as increases in food availability or cultural adaptations to support higher population sizes and densities, may lead to larger group sizes (S2). In these cases, there is evidence that as a group gets larger, it may also become more subdivided (S4; S7), either to reduce the social circles of individuals (S17), or to buffer against the spread of disease.

The unweighted networks were used as a less “noisy” test of our methods. We did, however, also test for associations between effective network sizes and parasite richness using weighted primate networks, which generally did not show improved performance over using raw network size. However, results from these analyses do indicate that effective network sizes estimated from

weighted networks were better predictors of parasite richness than unweighted networks. Additional factors complicating this relationship include, first, that social network structure more than likely has much greater intraspecific variation than other traits like body size, and the network structures presented in the literature may not be representative of the primate species which they represent. Additionally, the approach we used for our study, stochastic disease simulation, produces variability of its own, which may further obfuscate a relationship between structure and parasite risk.

3.5 References

S1. McCabe CM, Reader SM, Nunn CL. Infectious disease, behavioural flexibility and the evolution of culture in primates. Proc Biol Sci (2014) 282:20140862. doi:10.1098/rspb.2014.0862

S2. Wrangham RW. An ecological model of female-bonded primate groups. Behaviour (1980) 75:262-300.

S3. Nunn CL, Altizer SM. Infectious diseases in primates: behavior, ecology, and evolution. New York: Oxford University Press (2006).

S4. Nunn CL, Jordán F, McCabe CM, Verdolin JL, Fewell JH. Infectious disease and group size: more than just a numbers game. Philos Trans R Soc Lond B Biol Sci (2015) 370:20140111–20140111. doi:10.1098/rstb.2014.0111

S5. Rifkin JL, Nunn CL, Garamszegi LZ. Do animals living in larger groups experience greater parasitism? A meta-analysis. Am Nat (2012) 180:70–82. doi:10.1086/666081

S6. Nunn CL, Altizer SM, Jones KE, Sechrest W. Comparative tests of parasite species richness in primates. Am Nat (2003) 162:597–614. doi:10.1086/378721

S7. Griffin RH, Nunn CL. Community structure and the spread of infectious disease in primate social networks. Evol Ecol (2012) 26:779–800. doi:10.1007/s10682-011-9526-2

S8. Arnold C, Matthews LJ, Nunn CL. The 10kTrees website: A new online resource for primate phylogeny. Evol Anthropol (2010) 19:114–118. doi:10.1002/evan.20251

S9. Corbet GB, Hill JE. A World List of Mammalian Species. New York: Oxford University Press (1991).

S10. Paradis E, Claude J, Strimmer K. APE: Analyses of Phylogenetics and Evolution in R language. Bioinformatics (2004) 20:289. doi:10.1093/bioinformatics/btg412

S11. Nunn CL, Altizer SM. The global mammal parasite database: An online resource for infectious disease records in wild primates. Evol Anthropol (2005) 14:1–2. doi:10.1002/evan.20041

S12. Orme D, Freckleton R, Thomas G, Petzoldt T, Fritz S, Isaac N, Pearse W. caper: Comparative Analyses of Phylogenetics and Evolution in R. https://R-project.org/package=caper (2013)

S13. Mundry R. Statistical Issues and Assumptions of Phylogenetic Generalized Least Squares. (2014). doi:10.1007/978-3-662-43550-2_6

S14. Barton K. MuMIn: Multi-Model Inference. https://CRANR-projectorg/package=MuMIn (2016)

S15. Loehle C. Social Barriers to Pathogen Transmission in Wild Animal Populations. (1995). doi:10.2307/1941192

S16. VanderWaal KL, Atwill ER, Hooper S, Buckle K, McCowan B. Network structure and prevalence of Cryptosporidium in Belding's ground squirrels. Behavioral Ecology and Sociobiology. (2013). doi:10.1007/s00265-013-1602-x

S17. Dunbar RIM. Neocortex size as a constraint on group size in primates. J Hum Evol (1992) 22:469-493.
